# Reproducibility Evaluation of Urinary Peptide Detection Using CE-MS

**DOI:** 10.3390/molecules26237260

**Published:** 2021-11-30

**Authors:** Emmanouil Mavrogeorgis, Harald Mischak, Agnieszka Latosinska, Justyna Siwy, Vera Jankowski, Joachim Jankowski

**Affiliations:** 1 Mosaiques Diagnostics GmbH, 30659 Hannover, Germany; mavrogeorgis@mosaiques-diagnostics.com (E.M.); mischak@mosaiques-diagnostics.com (H.M.); latosinska@mosaiques.de (A.L.); siwy@mosaiques.de (J.S.); 2 Institute for Molecular Cardiovascular Research (IMCAR), RWTH Aachen University Hospital, 52074 Aachen, Germany; vjankowski@ukaachen.de; 3 Experimental Vascular Pathology, Cardiovascular Research Institute Maastricht (CARIM), University of Maastricht, 6229 Maastricht, The Netherlands

**Keywords:** biomarker, capillary electrophoresis, mass spectrometry, peptides, proteomics, urine

## Abstract

In recent years, capillary electrophoresis coupled to mass spectrometry (CE-MS) has been increasingly applied in clinical research especially in the context of chronic and age-associated diseases, such as chronic kidney disease, heart failure and cancer. Biomarkers identified using this technique are already used for diagnosis, prognosis and monitoring of these complex diseases, as well as patient stratification in clinical trials. CE-MS allows for a comprehensive assessment of small molecular weight proteins and peptides (<20 kDa) through the combination of the high resolution and reproducibility of CE and the distinct sensitivity of MS, in a high-throughput system. In this study we assessed CE-MS analytical performance with regards to its inter- and intra-day reproducibility, variability and efficiency in peptide detection, along with a characterization of the urinary peptidome content. To this end, CE-MS performance was evaluated based on 72 measurements of a standard urine sample (60 for inter- and 12 for intra-day assessment) analyzed during the second quarter of 2021. Analysis was performed per run, per peptide, as well as at the level of biomarker panels. The obtained datasets showed high correlation between the different runs, low variation of the ten highest average individual log2 signal intensities (coefficient of variation, CV < 10%) and very low variation of biomarker panels applied (CV close to 1%). The findings of the study support the analytical performance of CE-MS, underlining its value for clinical application.

## 1. Introduction

Capillary electrophoresis coupled to mass spectrometry (CE-MS) is a proteomic platform that has been extensively employed as a tool for clinical application, mainly for the non-invasive assessment of biomarkers in urine [1,2,3]. The approach relies on several steps leading to the biomarker identification and biomarker model construction. Before this can happen there are several quality control stages to ensure successful completion of each analysis, as described in detail in numerous review articles [1,3,4] and summarized below.

The technology consists of the CE-induced separation of analytes in a glass capillary in a high voltage field, followed by analyte ionization and analysis in MS, with the latter assessing the analyte molecular mass and relative abundance [5]. Separation is performed based on analyte electrophoretic mobility, determined by its charge and size. Since analytes may interact with silanol groups in the wall of the capillaries, thus interfering with separation, different capillary coating approaches were investigated to prevent this interaction [6]. Although coating could be of major benefit, current approaches failed to demonstrate the stability and reproducibility required for clinical application [7]. Therefore, the analysis is typically performed at low pH, where interaction of peptides with the capillary wall is kept to a minimum. CE is interfaced with MS instruments either sheathlessly or using sheath flow coupling, with the latter being commonly applied for analyzing samples in clinical context due to its higher reproducibility. Before entering the MS, analytes are ionized using electrospray ionization (ESI), similarly as in classic shotgun proteomics approach, where liquid chromatography (LC) is used as the separation technique, prior to MS analysis. Both of these separation techniques, CE and LC, are used in proteomics analysis, with LC more frequently applied, in part since it is simpler to operate and complete LC-MS solutions can be purchased. In comparison to LC, CE offers fast separation at high resolution with a stable buffer content (avoiding buffer gradients that may hamper detection by MS). A further benefit is that the capillary can be reconditioned after each analytical run at high pH (e.g., 0.1 M NaOH). This results in the strong robustness and reproducibility of CE. LC is less reproducible, mainly due to the fact that the LC column can never be fully reconditioned, resulting in the built-up of material (especially hydrophobic compounds) in the column that cannot be fully removed. Therefore, a substantial amount of carry-over is frequently observed, resulting in the detection of peptides/proteins from previous samples appearing in the currently analyzed sample. A negative point of CE is the small volume of the sample that can be loaded onto the capillary. This decreases the limit of detection in comparison to LC. Thus, LC may be the method of choice for in-depth analysis of very complex samples, while CE appears a better choice when robustness and reproducibility are required, as in the context of clinical application [8,9]. Based on the above, CE-MS is very well suited for the detection of naturally occurring peptides and small proteins (<20 kDa, termed peptidome), combining the superior separation capacity and reproducibility of CE with the high sensitivity of MS.

To assess information on the peptidome content in a sample, MS data need to be properly evaluated. In the past, a de novo approach was typically followed for the evaluation of CE-MS data, allowing defining lists of features (peptides) for each individual sample separately [10]. This approach was not only mathematically demanding, but also prone to errors due to the inherent difficulty of appointing the correct charge and identifying the monoisotopic peak. Therefore, a new concept was recently implemented: detected features are mapped onto a well-defined dataspace of, in the case of human urine, 21,559 peptides developed based on the analysis of more than 16,000 datasets [1]. Although, this approach does not allow the detection of novel compounds, it is characterized by improved consistency and lower variability as well as enhanced capacity of assessing lower quality signals, while being less computationally demanding and providing datasets of higher comparability. CE-MS on its own does not allow assigning the peptide sequence, which is required to link identified biomarkers with disease pathophysiology. The latter can be obtained by performing tandem mass spectrometry (MS/MS), either in combination with CE or LC, as described e.g., in Klein et al. [9].

Peptides indicative of disease (biomarkers) are detected through the comparison of datasets using statistical approaches, such as described previously in Dakna et al. and Stanley et al. [11,12]. Therefore, high comparability between datasets is required. This is achieved through the calibration of mass, migration time and signal intensity against internal standards for peptides consistently detected across multiple samples, as described by Jantos-Siwy et al. [13]. Calibration of mass and migration time allows for a consistent detection of the same peptides in multiple samples, while normalization of peptide intensities allows accounting for variation in sample concentration, due to factors like different fluid intake, diet, hormonal changes etc., which is especially relevant when analyzing urine. Defined biomarkers are used for the development of biomarker panels through their combination using support vector machines. Such panels have higher accuracy and stability than single biomarkers [10,14]. In addition, performance of the panel generally increases with the number of biomarkers included in the panel [4].

After more than a decade of utilizing and improving the aforementioned CE-MS workflow, the application of standard operating procedures (SOPs) and quality controls at each step of the analytical pipeline has led to the generation of the Human Urinary Proteome Database with over 85,000 CE-MS-based comparable urinary peptidomics profiles [1] with focus on kidney disease, cardiovascular disease, diabetes, oncology and transplantation, as well as healthy controls. Due to the good comparability of the datasets, the database can be used for assessment of the performance of developed biomarkers, eliminating the need for repeated measurements of control samples, assuring at the same time the statistical power.

Applications of CE-MS have been successfully utilized from chronic kidney disease (CKD) [15], cardiovascular disease [16] including heart failure [17] to cancer [3], as summarized in a recent review article [1]. Recently, CE-MS has been also applied in the context of SARS-CoV-2 [18,19,20]. Given the fact that urinary peptides are reflective of the health status of individuals, CE-MS has been applied mainly in the context of biomarker research, to define biomarkers for early detection of disease [21], prediction of disease progression [22,23], treatment response and patient stratification in the clinical trial [24]. Among the most recent applications, a proof-of-principle study for the use of CE-MS in the drug repurposing field was published, showing the potential of sodium/glucose cotransporter 2 inhibitors to combat COVID-19 [25]. In addition to reports on clinical applications, investigations of the technical aspects concerning robustness and comparability with other techniques have been performed [4,9], supporting the applicability of CE-MS as a tool for clinical purposes.

Since the last description of technical aspects and laboratory variability approximately a decade ago [4,26], several potential improvements were developed [1]. Among them, a substantial new implementation regarding the deconvolution of MS data, replacing the former de novo approach of defining peptides separately for every sample with a strategy that relies on a well-defined dataspace of peptides. Thus, the aforementioned dataspace in combination with the higher resolution of the instruments nowadays, leads to more consistent and a larger number of identified peptides. Consequently, our goal was to investigate the impact of these potential improvements on the reproducibility and robustness of this technique, analyzing results derived from 60 randomly selected runs of a well-characterized standard human urine sample analyzed based on our SOPs and quality control (QC) steps every second day [26]. Such a standard urine sample is representative of the urinary peptide content in healthy individuals and thus is used as a routine quality control for CE-MS instruments, allowing an assessment of pre-analytical steps, platform performance and data processing. The 60 runs served as the basis for the inter-day stability examination, while additional 12 runs analyzed within the same day were used for the assessment of intra-day stability. The present study evaluates technical aspects of CE-MS including among others reproducibility, variability and efficiency in peptide detection, at the level of individual run, single peptide or biomarker panel along with further characterization of peptidome content of the standard urine.

## 2. Results

### 2.1. Detection of Naturally Occurring Peptides in the Standard Urine Sample

**Figure 1:**
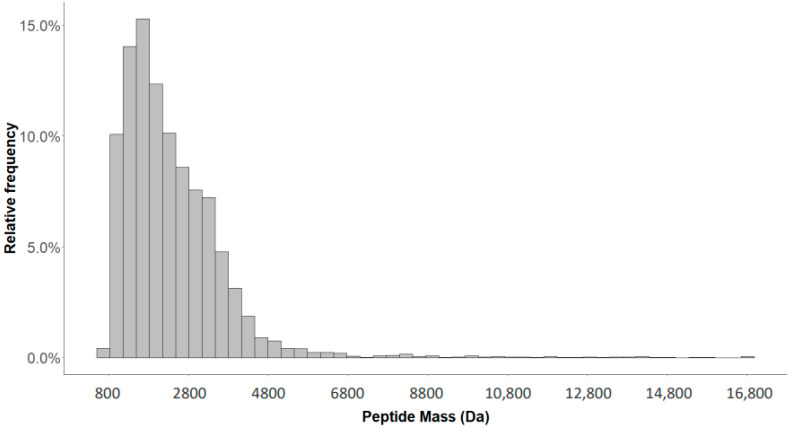
Distribution of the 13,547 peptide masses detected in standard urine sample.

Based on the acquired CE-MS data of 60 measurements of a standard human urine sample, a total of 13,547 different peptides were detected, defined by mass, CE migration time and signal intensity. The mass range of the detected peptide was 801.36–16,963.46. The observed normal distribution of the masses is illustrated in Figure 1.

Both the number of detected peptides and total log2 signal intensity were comparable between runs, with an average value of 4566 [95% confidence interval (CI): 4413 to 4717] and 31,433.5 (95% CI: 30,533.6 to 32,333.4), respectively. The descriptive statistics based on the log2 signal intensities of the detected peptides were also calculated for each run (Supplementary Table S1).

**Figure 2:**
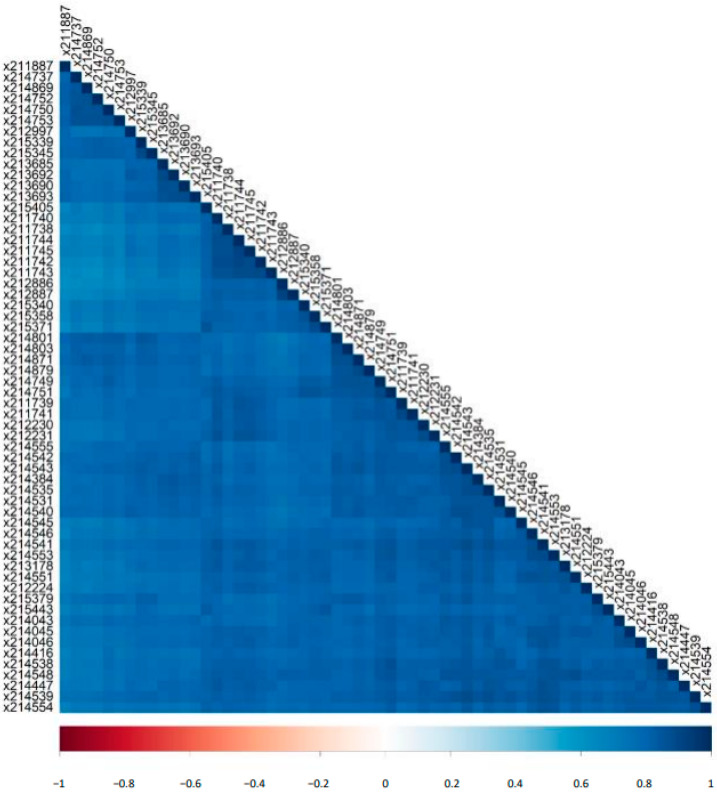
Correlation matrix for all detected peptides in the human standard urine sample. Shown are the correlation coefficients of the log2 signal intensities between the 60 different CE-MS runs.

We investigated the correlation of log2 signal intensities of all detected peptides between the 60 individual runs. The resulted correlation matrix based on the Pearson correlation coefficient (Pearson’s r) is shown in Figure 2. Observed was a high and significant (*p*-value < 0.05) correlation between the runs with a mean Pearson’s r of 0.79 (±0.05).

### 2.2. Variability of Signal Intensities of Individual Peptides

The variation in CE-MS log2 signal intensity of individual peptides was investigated. The analysis focused on the ten most abundant peptides defined based on the highest average log2 signal intensities in 60 measurements (inter-day variability) as well as in 12 additional measurements (intra-day variability). The descriptive statistics are presented in Table 1.

**Figure 3:**
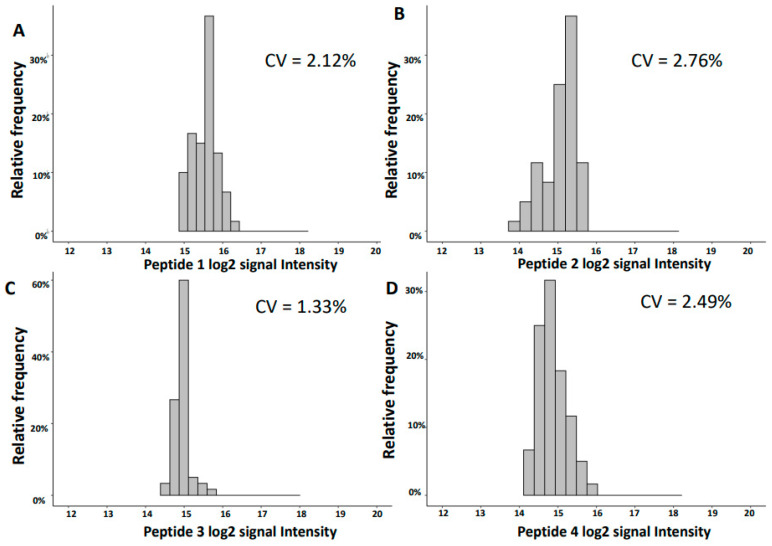
Distributions of the log2 intensities of the four most abundant peptides based on mean log2 signal intensity. (**A**) SGSVIDQSRVLNLGPIT (Uromodulin). (**B**) NTGAPGSpGVSGpKGDAGQpGEKGSpGAQGppGAPGPLG (Collagen alpha-1(III)). (**C**) GGpGSDGKPGppGSQGESGRPGPpG (Collagen alpha-1(III)). (**D**) DEAGSEADHEGTHSTKRG (Fibrinogen alpha). p: Hydroxyproline. CV: coefficient of variation.

The ten most abundant peptides detected are fragments of uromodulin, collagen alpha-1(III), fibrinogen alpha, collagen alpha-1(I) and collagen alpha-1(IV). The mean log2 signal intensities for the inter-day reproducibility ranged from 14.04 to 15.56, while their coefficients’ of variation (CV) range was 1.3–5.7%, supporting good reproducibility. The distributions of the log2 signal intensities of the top four most abundant peptides along with the respective CV are shown in Figure 3.

The mean log2 signal intensities observed in the 12 analyses performed within one day did not show significant differences in comparison to the mean log2 intensities for the 60 runs described above (*p*-value = 0.403). As expected, the CV were significantly lower (*p*-value = 0.007) within one day in comparison to inter-day analyses and ranged from 0.7% to 1.6%.

When comparing the ten most abundant peptides with other reports on urinary peptides in the literature, we observed a very good overlap. Eight of these ten peptides were also found to be among the top forty urinary peptides detected in human female and male standard urine samples using CE- and LC-MS/MS, as described in 2010 [26]. For the two peptides that were not reported in the previous study (fragments of collagen alpha-1(III) and collagen alpha-1(IV)), information on their sequence was obtained after 2010. Seven of the top ten peptides were also found in a study by Kononikhin et al. [27] investigating the urine samples of pregnant women by LC-MS/MS. Van Huizen et al. [28] performed LC-MS/MS analysis of urine samples from healthy controls (*n* = 100) and patients with colorectal cancer developing liver metastases (*n* = 100). Six of the top ten peptides presented in our study were also identified in this work. In addition, five of the most abundant peptides were also reported in a study by Van et al. [29]. Of note, the smaller overlap may be due to the low number of urine samples analyzed in the study (i.e., *n* = 30, 15 of healthy controls and 15 of patients with type I diabetes). The two uromodulin fragments within the top ten peptides reported here were consistently found in all presented studies. These uromodulin peptides do not contain any post-translational modifications, easing their identification. Identification of peptides containing post-translational modification (like in the case of collagen fragments that contain proline hydroxylation) obviously mandates including the modification as variable term in the search. If this is not implemented, then the peptide cannot be detected. This is, most likely, the reason why some publications on urine peptides fail to detect collagen-derived peptides: the authors were unaware of the requirement to account for proline hydroxylation.

### 2.3. Variability of Biomarker Panels

**Figure 4:**
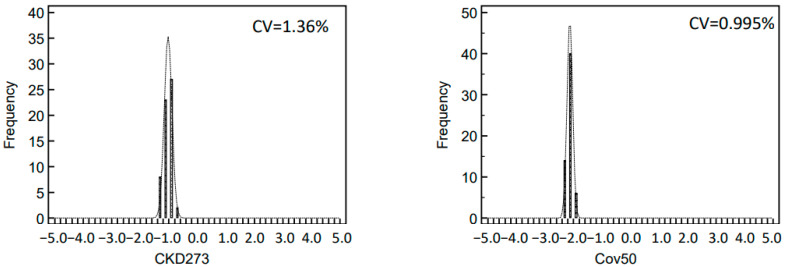
Distribution of classification scores of selected biomarker panels. The left histogram depicts the classification scores of CKD273 for 60 measurements of the standard human urine sample. The right histogram depicts the classification score of Cov50 biomarker panel for the same set of measurements. None of the measurements scored positive in either panel. In addition, the coefficient of variation (CV) related to classification score range is given.

Given the application of the biomarker panels in clinical practice, we also investigated the variability of the classification scores for two selected panels based on urinary peptides. As examples we selected the CKD273 biomarker panel applied for early diagnosis of CKD [30] and the Cov50 biomarker panel for prediction of worse outcomes in SARS-CoV-2-infected patients [18]. Both panels were developed using support vector machines and were applied to classify the 60 runs of the standard human urine sample. The distribution of the scores for both biomarkers panels is shown in Figure 4. The diagnostic threshold for both biomarker panels was shifted to 0 to allow easier comparison of the results. Samples scoring positive are indicative of the respective disease. The score dataspace is from −5 (negative scores) to +5 (positive scores). As expected, all datasets from the analysis of the standard urine sample, being representative for healthy individuals, scored negative for both panels. Within this range from −5 to +5, the resulted CV was 1.36% for the CKD273 and 0.995% for Cov50.

### 2.4. Characterization of the Urinary Peptidome Representative of a Healthy Individual

**Figure 5:**
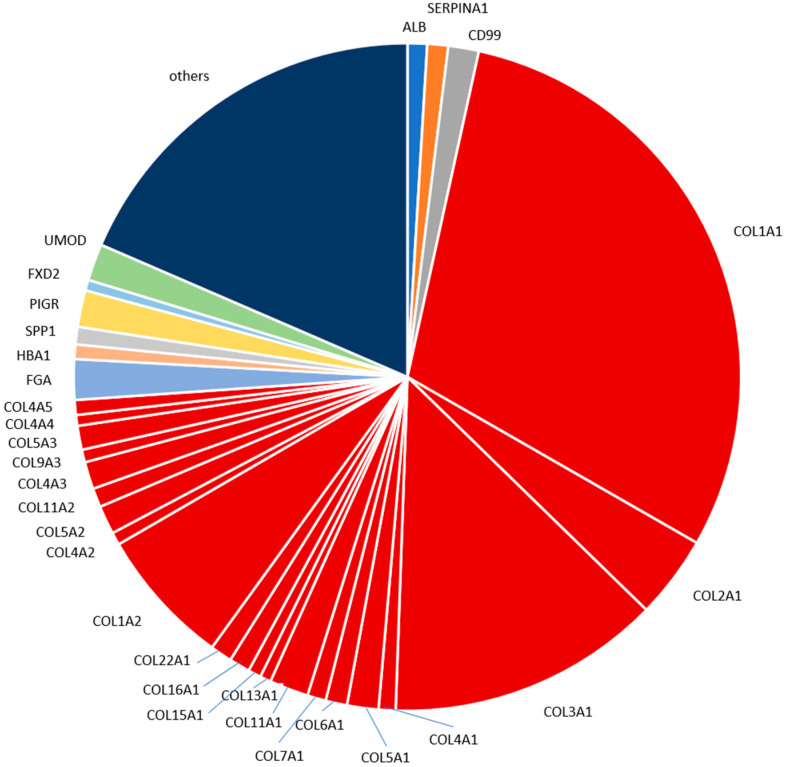
Pie chart depicting the proteome of the standard urine sample. Shown are the top thirty abundant proteins detected in the standard human urine sample. Red-colored areas of the charts indicate collagen proteins. Abbreviations can be found in Supplementary Table S2.

The identified peptides detected based on 60 measurements of the standard human urine sample represent fragments of 389 different proteins. Information on peptide sequence, average peptide log2 signal intensity in all measurements and the respective parental protein were used to better characterize the content of the standard urine sample. Figure 5 presents the distribution of the top thirty most abundant parental proteins that gave rise to the identified urinary peptides. For those, protein abundance was calculated based on the sum of the log2 intensities of individual peptides belonging to the respective protein. As shown, collagen fragments are responsible for the majority (75%) of urinary peptides in healthy adults, with collagen alpha-1(I), collagen alpha-1(III) and collagen alpha-2(I) being the most abundant members of the collagen family. The high abundance of collagen-derived peptides might be related to the high abundance of collagen in the human body and due to the possible exclusion of these peptides from tubular reabsorption [31]. Other peptides represent proteins most likely derived from blood (fibrinogen alpha chain, alpha-1-antitrypsin, albumin), and kidney (e.g., uromodulin, polymeric immunoglobulin receptor). 

## 3. Discussion

An increasing number of reports underlines the clinical value of urinary peptidomic analysis using CE-MS. A focus of the application has been kidney disease, which, among others, has resulted in a letter of support from the US FDA [32] (https://www.fda.gov/media/98846/download, accessed on 27 November 2021) for this approach. Also a major step forward was the successful completion of PRIORITY [21], the first multicenter randomized controlled trial to apply CE-MS for patient stratification. CE is a simple separation method that allows high resolution separation of analytes. The direct comparison of CE with LC showed much lower variability of CE-MS based data [26]. Reproducibility of CE-MS has been assessed previously [4]. However, the reproducibility depends not only on the CE-MS analysis itself, but also on sample preparation, data calibration, and data handling. Thus, SOPs need to be rigorously followed and any changes in any of the mentioned steps may impact the reproducibility. A major update in our current work-flow includes the deconvolution of MS data: the former de novo approach of defining peptides separately for every sample was replaced by a strategy that relies on a well-defined dataspace of peptides. The aim of this study was to investigate the reproducibility of CE-MS based on data obtained using our current methods for sample analysis and data evaluation [1]. Towards this direction, we used randomly selected 60 and additional 12 measurements of one standard human urine sample to analyze the inter- and intra-day reproducibility, respectively. Good reproducibility between the different runs in the number and total abundance of the detected peptides was observed. The peptide log2 signal intensities correlated well between the different runs. We also detect very good overlap with previous reports, either from our or other groups. Almost all of the most abundant urinary peptides detected in the analyzed standard human urine sample were also found in previously published studies.

The observed variation of the log2 signal intensities between the different runs was low with CV for inter-day reproducibility in the range of 1.3–5.7% and for the intra-day of 0.7–1.6% for the ten most abundant peptides. This low variability is of major benefit in statistical analysis when defining disease-specific biomarkers. The variation of the data is further reduced when combining individual biomarkers into a panel, as exemplified by the investigation of CKD273 and the recently developed Cov50 biomarker panels. The CV of both investigated biomarker panels was lower than the CV of individual peptides, even those of high abundance.

The most abundant peptides detected in the human standard urine sample in our analysis are derived from proteins well expected and already reported to be present in urine, namely different collagen types, fibrinogen alpha chain, uromodulin, polymeric immunoglobulin receptor, CD99 antigen and alpha-1-antitrypsin. For example, in a study focused on colorectal liver metastasis that employed LC-MS/MS [28], 27% of the identified naturally occurring peptides originated from different collagens; while the top three proteins with the highest number of identified fragments were collagen alpha-1(I), collagen alpha-1(III) and uromodulin. Our study also indicated a high contribution of collagen fragments in the standard urine sample proteome. Peptides from collagen alpha-1(I) and collagen alpha-1(III) were the two most frequent (and abundant), with peptides from uromodulin ranking as the 12th most frequent and 7th most abundant. The aforementioned differences may reflect the LC and CE relation, which has been reported as complementary [9]. In another LC-MS/MS based study investigating early type 1 diabetes [29], eight of the ten proteins that gave rise to the most frequently observed peptides namely, albumin, collagen alpha-1(I) chain, hemoglobin subunit alpha, hemoglobin subunit beta, apolipoprotein A-I, protein S100-A9, collagen alpha-2(I) chain, uromodulin, were also included in the seventeen most frequent proteins in our study. As for the ten most abundant peptides based on mean intensities presented in our study, a high overlap was observed with the respective reports by Mischak et al., Kononikhin et al., van Huizen et al. and Van et al. [26,27,28,29]. In all these reports, naturally occurring peptides were analyzed without prior digestion. Many of these peptides contain post-translational modifications. These modifications hinder the identification of the amino acid sequence. This may explain our observation that mainly larger collagen fragments containing multiple proline hydroxylations were not consistently observed in the different studies. The two uromodulin fragments of the top ten most abundant peptides detected here in the standard human urine sample were found in each of the four other reports as well. This is expected, since uromodulin is a highly abundant kidney protein excreted in urine. The two fragments originate from the C-terminus of uromodulin. The peptides from this region are typically detected by analysis of native peptides [26,29].

## 4. Conclusions

The CE-MS presents a robust and reproducible platform for peptide analysis to be applied in clinical proteomics. The development in the methods for sample preparation, sample analysis and data evaluation resulted in the generation of well comparable datasets (based on both inter-day and intra-day results) that can be now fully exploited for the definition of robust disease-specific biomarkers, specifically biomarker panels. At the same time, given the performance of the platform and available biomarker panels based on CE-MS analysis of urinary peptides, full advantage should be taken to fulfill the main goal of clinical proteomics, which is clinical implementation.

## 5. Methods

### 5.1. Urine Sample

The standard human urine sample used in this study was described in detail before [26]. It consists of a pooled midstream morning urine of 8 female healthy anonymous volunteers from multiple collections. Urine collections were performed without any requirements relevant to the diet or the menstrual cycle (only absence of menstruation) of the participants. The approach of pooled urine was followed since it is considered more practical and less technically demanding than individual urine. This urine collection protocol is in agreement to a “standard protocol for urine collection” developed by the Human Urine and Kidney Proteome Project and European Kidney and Urine Proteomics COST Action (EuroKUP) networks [26]. There was no addition of protease or phosphatase inhibitors or pH adjustment. The 40–100 mL collected samples were frozen immediately at −20 °C. Upon completion of collection, all frozen samples were thawed on ice, sonicated, combined, divided into several 1, 10, and 50 mL aliquots, and frozen again at −80 °C The urinary proteome is not affected significantly by up to three freeze/thaw cycles following initial freezing [33]. Measurements selected were of the second quarter of 2021 and included 60 for inter-day and 12 for intra-day investigation of the stability of the CE-MS analysis.

### 5.2. Capillary Electrophoresis Mass Spectrometry

Sample preparation and CE-MS analysis was performed according to SOP allowing for the recovery of over 80% [4,34]. In detail, urine samples were initially thawed and then diluted by mixing 700 μL with an aqueous solution of 700 μL of 2 M urea, 10 mM NH_4_OH containing 0.02% sodium dodecyl sulfate (SDS) to inhibit interactions between proteins. Afterwards, a Centristat 20 kDa cut-off centrifugal filter device (Sartorius, Göttingen, Germany) was utilized for the ultrafiltration of the samples to eliminate high molecular weight proteins. Subsequently, urea, salts and electrolytes were removed from the obtained filtrate by desalting using a PD 10 gel filtration column (GE Healthcare Bio Sciences, Uppsala, Sweden) equilibrated with 0.01% NH_4_OH in HPLC-grade water. Then, lyophilization and storage at 4 °C occurred, until the measurement of the samples using CE-MS.

Shortly before CE-MS analysis, the samples were resuspended in 10 μL HPLC-grade H_2_O. The analysis was performed using a P/ACE MDQ capillary electrophoresis system (Beckman Coulter, Fullerton, CA, USA) coupled to a micro-TOF-MS (Bruker Daltonic, Bremen, Germany). Before running each sample, the capillary was conditioned with NaOH (1 M in demineralized water) at pressure of 50 psi for 10 min was applied, followed by a final wash of the capillary with NH_4_OH solution (3.76 mL of ammonium solution (25%) made up to 50 mL with the addition of HPLC-grade water) at 50 psi for 10 min and 20 min with running buffer (a solution of 20% acetonitrile (Sigma-Aldrich, Taufkirchen, Germany) in HPLC-grade water supplemented with 0.94% formic acid (Sigma-Aldrich)). After this conditioning, the capillary (90 cm, 50 μm ID) was connected to the MS. The Beckmann CE parameters of the analysis software were set on ‘reverse’, when connected to the MS system using the external detector adapter (EDA), as the separation of the peptides was achieved by reverse polarity to match the polarity of the CE with that of the MS ionization voltage. Initially, the running buffer was rinsed into the capillary for 2 min at 50 psi, before the injection of each sample. Samples were then injected into CE-MS at 2 psi for 99 sec, resulting in injection volumes of ~290 nL. At the injection side of the capillary +25 kV were applied for 30 min, enabling separation of the analytes at a capillary set temperature of 35 °C. Then, along with the +25 kV separation voltage, pressure was applied (0.1 psi for 1 min, 0.2 psi for 1 min, 0.3 psi for 1 min, 0.4 psi for 1 min and 0.5 psi for 30 min). Coaxially, sheath liquid (15 mL of 2-propanol, 200 µl of formic acid with addition of HPLC-grade water until 50 mL) was applied with flow rates in the range of 0.02 mL/h (without nebulizer gas). For the CE-MS analysis, the electrospray ionization interface (Agilent Technologies, Palo Alto, CA, USA) was set to a potential of −4.0 to −5 kV and the ESI sprayer was grounded to achieve electric potential zero. Spectra were recorded over a *m*/*z* range of 400–3000 and accumulated every 3 sec for about 60 min. A resolution in the range of 10,000 characterized monoisotopic mass signals resolved for *z* ≤ 6.

### 5.3. MS Data Evaluation

The raw data of the CE-MS analysis are saved in the database to the corresponding clinical information based on the unique identification sample number. The evaluation of raw MS data was performed with MosaFinder software that is based on a probabilistic clustering algorithm and utilizing isotopic distributions and conjugated masses for charge state determination [1]. Twenty-nine collagen fragments, on which the disease, in general, has no impact, were used as internal standards for the normalization of the obtained signal intensities [13]. An in silico assignment of the identified fragments to sequenced peptides from the Human Urinary Proteome database was performed [1].

### 5.4. Statistics

The signal intensities of the detected peptides were log2 transformed after entries with zeros were converted into missing values. The results and findings of the current paper were based on R programming (R version 4.1.0, R Foundation Statistical Computing, Vienna, Austria) [35]. The descriptive statistics per run and per peptide were calculated with the function stat.desc of the R package pastecs. The Pearson correlation matrix was calculated with the rcorr function of Hmisc R package and generated as a plot with the R package corrplot. The plot for the mass distribution of the identified analytes as well as the plots of the distributions of the log2 intensities of the four most abundant peptides were created based on the package ggplot2 and the latter plots were arranged in the same figure using ggpubr R package. The plots of the distributions of classification scores were generated using MedCalc software (version 12.1.0.0; MedCalc Sofware, Mariakerke, Belgium). The latter software was also used to perform the parametric t-test for the comparison of inter- and intra-day results with respect to their mean log2 signal intensities.

## Figures and Tables

**Table 1:** Descriptive statistics of the ten most abundant peptides. Given is also information with regards to the peptide annotation, CE-MS properties and mean log2 signal intensity for both inter- and intra-day analyses. p: Hydroxyprolines. Int: Intensity.

					Inter-Day			Intra-Day	
Mass [Da]	CE Time [Min]	Sequence	Gene Symbol	Mean log2 Int	SD	CV [%]	Mean log2 Int	SD	CV [%]
1754.92	31.39	SGSVIDQSRVLNLGPIT	UMOD	15.56	0.33	2.12	15.48	0.17	1.07
3457.61	31.46	NTGAPGSpGVSGpKGDAGQpGEKGSpGAQGppGAPGPLG	COL3A1	15.05	0.42	2.76	15.29	0.18	1.18
2248.99	26.16	GGpGSDGKPGppGSQGESGRPGPpG	COL3A1	14.94	0.20	1.33	14.83	0.14	0.98
1882.80	20.24	DEAGSEADHEGTHSTKRG	FGA	14.87	0.37	2.49	14.64	0.13	0.91
2825.28	24.45	ERGEAGIpGVpGAKGEDGKDGSpGEpGANG	COL3A1	14.76	0.28	1.92	14.72	0.13	0.88
1250.56	28.00	ApGDRGEpGPPGp	COL1A1	14.65	0.18	1.26	14.34	0.11	0.75
2169.97	26.10	NSGEpGApGSKGDTGAKGEPGpVG	COL1A1	14.61	0.21	1.46	14.45	0.16	1.10
2047.92	21.93	NGDDGEAGKpGRpGERGPPGP	COL1A1	14.37	0.62	4.32	14.33	0.23	1.62
1911.05	25.23	SGSVIDQSRVLNLGPITR	UMOD	14.34	0.82	5.75	11.29	0.18	1.61
3441.61	31.36	DGAPGQKGEMGPAGPTGPRGFpGppGPDGLPGSMGPP	COL4A1	14.04	0.33	2.36	14.49	0.23	1.57

## Data Availability

Data will be made available upon request directed to the corresponding author. Proposals will be reviewed and approved by the investigators and collaborators based on scientific merit. After approval of a proposal, data will be shared through a secure online platform after signing a data access and confidentiality agreement.

## Supplementary Materials

The following are available online. Table S1: descriptive statistics of log2 60 runs. Table S2: Protein abbreviations for Figure 5.

## Sample Availability

The sample is freely available from the authors upon request.

## References

[1] Latosinska A., Siwy J., Mischak H., Frantzi M. (2019). Peptidomics and proteomics based on CE-MS as a robust tool in clinical application: The past, the present, and the future. Electrophoresis.

[2] Stalmach A., Albalat A., Mullen W., Mischak H. (2013). Recent advances in capillary electrophoresis coupled to mass spectrometry for clinical proteomic applications. Electrophoresis.

[3] Latosinska A., Frantzi M., Vlahou A., Mischak H. (2013). Clinical applications of capillary electrophoresis coupled to mass spectrometry in biomarker discovery: Focus on bladder cancer. Proteom. Clin. Appl..

[4] Mischak H., Vlahou A., Ioannidis J.P. (2012). Technical aspects and inter-laboratory variability in native peptide profiling: The CE–MS experience. Clin. Biochem..

[5] Mischak H., Schanstra J.P. (2010). CE-MS in biomarker discovery, validation, and clinical application. Proteom. Clin. Appl..

[6] Huhn C., Ramautar R., Wuhrer M., Somsen G.W. (2009). Relevance and use of capillary coatings in capillary electrophoresis–mass spectrometry. Anal. Bioanal. Chem..

[7] Pontillo C., Filip S., Borràs D.M., Mullen W., Vlahou A., Mischak H. (2015). CE-MS-based proteomics in biomarker discovery and clinical application. Proteom. Clin. Appl..

[8] Kolch W., Neusüß C., Pelzing M., Mischak H. (2005). Capillary electrophoresis-mass spectrometry as a powerful tool in clinical diagnosis and biomarker discovery. Mass Spectrom. Rev..

[9] Klein J., Papadopoulos T., Mischak H., Mullen W. (2014). Comparison of CE-MS/MS and LC-MS/MS sequencing demonstrates significant complementarity in natural peptide identification in human urine. Electrophoresis.

[10] Mischak H., Coon J.J., Novak J., Weissinger E.M., Schanstra J.P., Dominiczak A. (2008). Capillary electrophoresis-mass spectrometry as a powerful tool in biomarker discovery and clinical diagnosis: An update of recent developments. Mass Spectrom. Rev..

[11] Dakna M., Harris K., Kalousis A., Carpentier S., Kolch W., Schanstra J.P., Haubitz M., Vlahou A., Mischak H., Girolami M. (2010). Addressing the Challenge of Defining Valid Proteomic Biomarkers and Classifiers. BMC Bioinform..

[12] Stanley E., Delatola E.I., Nkuipou-Kenfack E., Spooner W., Kolch W., Schanstra J.P., Mischak H., Koeck T. (2016). Comparison of different statistical approaches for urinary peptide biomarker detection in the context of coronary artery disease. BMC Bioinform..

[13] Siwy J., Schiffer E., Brand K., Schumann G., Rossing K., Delles C., Mischak H., Metzger J. (2008). Quantitative Urinary Proteome Analysis for Biomarker Evaluation in Chronic Kidney Disease. J. Proteome Res..

[14] Rodríguez-Suárez E., Whetton A.D. (2012). The application of quantification techniques in proteomics for biomedical research. Mass Spectrom. Rev..

[15] Mavrogeorgis E., Mischak H., Beige J., Latosinska A., Siwy J. (2021). Understanding glomerular diseases through proteomics. Expert Rev. Proteom..

[16] Brown C.E., McCarthy N., Hughes A., Sever P., Stalmach A., Mullen W., Dominiczak A., Sattar N., Mischak H., Thom S. (2015). Urinary proteomic biomarkers to predict cardiovascular events. Proteom. Clin. Appl..

[17] He T., Mischak M., Clark A.L., Campbell R.T., Delles C., Díez J., Filippatos G., Mebazaa A., McMurray J.J., González A. (2021). Urinary peptides in heart failure: A link to molecular pathophysiology. Eur. J. Heart Fail..

[18] Wendt R., Thijs L., Kalbitz S., Mischak H., Siwy J., Raad J., Metzger J., Neuhaus B., von der Leyen H., Dudoignon E. (2021). A urinary peptidomic profile predicts outcome in SARS-CoV-2-infected patients. EClinicalMedicine.

[19] Wendt R., Kalbitz S., Lübbert C., Kellner N., Macholz M., Schroth S., Ermisch J., Latosisnka A., Arnold B., Mischak H. (2020). Urinary Peptides Significantly Associate with COVID-19 Severity: Pilot Proof-of-Principle Data and Design of a Multicentric Diagnostic Study. Proteomics.

[20] Siwy J., Wendt R., Albalat A., He T., Mischak H., Mullen W., Latosinska A., Lübbert C., Kalbitz S., Mebazaa A. (2021). CD99 and polymeric immunoglobulin receptor peptides deregulation in critical COVID-19: A potential link to molecular pathophysiology?. Proteomics.

[21] Tofte N., Lindhardt M., Adamova K., Bakker S.J.L., Beige J., Beulens J.W.J., Birkenfeld A.L., Currie G., Delles C., Dimos I. (2020). Early detection of diabetic kidney disease by urinary proteomics and subsequent intervention with spironolactone to delay progression (PRIORITY): A prospective observational study and embedded randomised placebo-controlled trial. Lancet Diabetes Endocrinol..

[22] Pontillo C., Zhang Z.-Y., Schanstra J.P., Jacobs L., Zürbig P., Thijs L., Ramírez-Torres A., Heerspink H.J., Lindhardt M., Klein R. (2017). Prediction of Chronic Kidney Disease Stage 3 by CKD273, a Urinary Proteomic Biomarker. Kidney Int. Rep..

[23] Pontillo C., Jacobs L., Staessen J.A., Schanstra J.P., Rossing P., Heerspink H.J., Siwy J., Mullen W., Vlahou A., Mischak H. (2016). A urinary proteome-based classifier for the early detection of decline in glomerular filtration. Nephrol. Dial. Transplant..

[24] Lindhardt M., Persson F., Oxlund C., Jacobsen I.A., Zürbig P., Mischak H., Rossing P., Heerspink H.J. (2017). Predicting albuminuria response to spironolactone treatment with urinary proteomics in patients with type 2 diabetes and hypertension. Nephrol. Dial. Transplant..

[25] Latosinska A., Siwy J., Cherney D.Z., Perkins B.A., Mischak H., Beige J. (2021). SGLT2-Inhibition reverts urinary peptide changes associated with severe COVID-19: An in-silico proof-of-principle of proteomics-based drug repurposing. Proteomics.

[26] Mischak H., Kolch W., Aivaliotis M., Bouyssié D., Court M., Dihazi H., Dihazi G.H., Franke J., Garin J., de Peredo A.G. (2010). Comprehensive human urine standards for comparability and standardization in clinical proteome analysis. Proteom. Clin. Appl..

[27] Kononikhin A.S., Zakharova N.V., Sergeeva V.A., Indeykina M.I., Starodubtseva N.L., Bugrova A.E., Muminova K.T., Khodzhaeva Z.S., Popov I.A., Shao W. (2020). Differential Diagnosis of Preeclampsia Based on Urine Peptidome Features Revealed by High Resolution Mass Spectrometry. Diagnostics.

[28] Van Huizen N.A., Van Rosmalen J., Dekker L.J.M., Braak R.R.J.C.V.D., Verhoef C., Ijzermans J.N.M., Luider T.M. (2019). Identification of a Collagen Marker in Urine Improves the Detection of Colorectal Liver Metastases. J. Proteome Res..

[29] Van J.A.D., Clotet-Freixas S., Zhou J., Batruch I., Sun C., Glogauer M., Rampoldi L., Elia Y., Mahmud F.H., Sochett E. (2020). Peptidomic Analysis of Urine from Youths with Early Type 1 Diabetes Reveals Novel Bioactivity of Uromodulin Peptides In Vitro. Mol. Cell. Proteom..

[30] Good D.M., Zürbig P., Argilés A., Bauer H.W., Behrens G., Coon J.J., Dakna M., Decramer S., Delles C., Dominiczak A. (2010). Naturally Occurring Human Urinary Peptides for Use in Diagnosis of Chronic Kidney Disease. Mol. Cell. Proteom..

[31] He T., Pejchinovski M., Mullen W., Beige J., Mischak H., Jankowski V. (2020). Peptides in Plasma, Urine, and Dialysate: Toward Unravelling Renal Peptide Handling. Proteom. Clin. Appl..

[32] Pontillo C., Mischak H. (2017). Urinary peptide-based classifier CKD273: Towards clinical application in chronic kidney disease. Clin. Kidney J..

[33] Thongboonkerd V., Mungdee S., Chiangjong W. (2009). Should Urine pH Be Adjusted Prior to Gel-Based Proteome Analysis?. J. Proteome Res..

[34] Theodorescu D., Fliser D., Wittke S., Mischak H., Krebs R., Walden M., Ross M., Eltze E., Bettendorf O., Wülfing C. (2005). Pilot study of capillary electrophoresis coupled to mass spectrometry as a tool to define potential prostate cancer biomarkers in urine. Electrophoresis.

[35] Wickham H. (2016). Ggplot2: Elegant Graphics for Data Analysis.

